# Improving Maternal and Child Health in Difficult Environments: The Case For “Cross-Border” Health Care

**DOI:** 10.1371/journal.pmed.1000005

**Published:** 2009-01-13

**Authors:** Gijs Walraven, Semira Manaseki-Holland, Abid Hussain, John B Tomaro

## Abstract

Gijs Walraven and colleagues discuss maternal and child health programs in adjacent geographical areas in difficult environments in Afghanistan, Pakistan, and Tajikistan.

The evidence base on organising, delivering, and paying for effective and equitable health services in any resource-constrained setting is very weak [1]. To make health service delivery work in difficult environments, such as Badakhshan province in Afghanistan, the Northern Areas of Pakistan, and Gorno-Badakhshan province in Tajikistan, substantial challenges must be addressed because each has to overcome one or more “traps” of bad governance, emerging from civil war, and/or being landlocked [2]. To improve maternal and child health (MCH), emphasis must be given to strengthening health systems, increasing access to information and care, and addressing the related community and development issues [3].

In the high mountainous areas of Central Asia, the successor states of the Russian and British empires govern communities that are often forgotten and frequently unserved [4]. In some measure, the health status of these communities, especially that of the women and children living in the contiguous border areas of three states (Afghanistan, Pakistan, and Tajikistan) reflects the challenges, successes, and failures of these states. Although the communities in these adjacent geographical areas share a common ethnicity, religion, and culture, MCH indicators vary widely along with the capacities and efforts of governmental and non-governmental actors to reduce the disparities.

In the rural province of Afghan Badakhshan, a remote region with minimal infrastructure and few modern health services, Linda Bartlett and others recently carried out a reproductive age mortality survey [5]. Reported in 2005, this survey found the highest maternal mortality ratio ever documented (6,507 per 100,000 live births) for a three-year time period (April 1999 through March 2002), and a very high infant mortality rate (217 per 1,000 live births) [5]. Gorno-Badakhshan Autonomous Oblast sits on the other side of the Oxus (Amu Darya, Panji) River in the newly independent Republic of Tajikistan. Here, the Soviet health system contributed to a relatively low maternal mortality ratio (54 per 100,000 live births) and infant mortality rate (28 per 1,000 live births), even as late as 1994. Recent surveys in the Oblast suggest that the new republic's health system has not been able to maintain or improve the indicators achieved during the Soviet period. In the Northern Areas of Pakistan, a disputed territory but contiguous with the other regions, local government institutions have traditionally been very weak. However, important improvements in maternal and child health indicators, including a substantial reduction in the maternal mortality ratio (from 550 to 68 per 100,000 live births) and infant mortality rate (from 158 to 31 per 1,000 live births) have been observed over the last 20 years. While under-reporting of maternal, infant, and especially neonatal deaths is a global problem, and variations in data collection methods challenge the comparability of the measures across the three regions, the substantial differences strongly suggest true distinctions that should be examined to determine why women and infants in the Northern Areas and Gorno-Badakhshan have markedly lower risk of death.

**Summary Points**

- Health indicators, including levels of maternal and infant mortality, are very different in adjacent geographical border areas of Afghanistan, Pakistan, and Tajikistan.
- These differences reflect the combined and complex interplay of elements within the different health systems, as well as political, economic, social, and cultural factors.
- Reducing maternal and child mortality requires focus and balance in all of these dimensions and can best be achieved through service interventions underpinned by general development.
- A policy promoting “cross-border” health programmes could immediately make available existing resources that could contribute to reducing maternal and child mortality in all three geographical locations.

## MCH in Afghan Badakhshan

The challenges facing the Afghanistan health authorities in the province of Badakhshan are daunting. The population is poor, largely illiterate, and widely dispersed, and health facilities are few, unequipped, and staffed by a small number of poorly trained staff (see Table 1). The Afghan government, with support from international donor agencies, contracted the Aga Khan Development Network (AKDN; http://www.akdn.org/) to implement a health programme in the province. The programme, which started in 2003, follows the Ministry of Public Health policy that makes a single nongovernmental organisation responsible for planning, implementing, and monitoring the health services in a district or province. This approach attempts to avoid duplication of effort, ensures efficient control of resources, and promotes effective programme management. Care is provided through a three-tiered system, consisting of:

1. community health workers (CHWs); one woman and one man per community of around 1,000 people who are chosen by the community, native to the local area, and literate where possible;
2. two to four basic health centres with outreach (outpatient services and normal deliveries, covering a population of 5,000–10,000); and
3. one comprehensive referral health centre covering a minimum population of 25,000 (limited inpatient capacity, but in border areas of Badakhshan includes complete essential obstetric services as well as blood transfusion services) or district hospital per cluster [6].

**Table 1:**
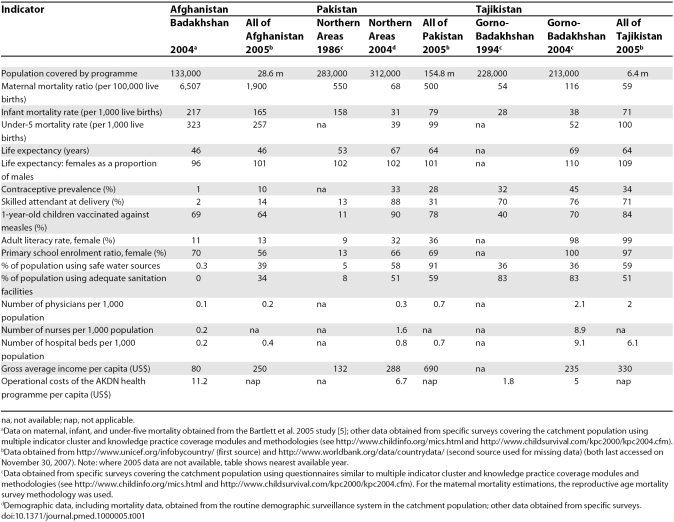
Health Indicators for Afghanistan and Afghan Badakhshan, Pakistan and the Northern Areas, and Tajikistan and Gorno-Badakhshan

At the close of 2006, the system in the Badakhshan border districts included 140 CHWs in 70 villages, seven basic health centres, and three comprehensive referral health centres. This network is providing health care to an estimated 133,000 people, and focuses on MCH interventions such as child immunisation; micronutrient supplementation and nutrition screening; prenatal, delivery, and post-partum care; family planning; and basic curative services, including integrated management of childhood illnesses. A community midwifery school, affiliated with the provincial hospital, has been established. In September 2006, the first 18 graduates, who received 18 months of training that included modules on child health care and health promotion and prevention, were posted at health centres closest to their home communities.

In addition to health services, other interventions are being implemented. Interventions in education (with a major emphasis on female education), natural resource management, agriculture and marketing, water and sanitation infrastructure, road construction, telecommunication, electricity, microfinance, small business development, and civil society promotion are combined with economic development and cultural restoration activities to constitute an area development programme. The impact of this approach to reducing maternal and child mortality remains to be determined, along with an investigation to identify the components with the most significant impact. Still, there are promising early signs of increases in the availability and utilisation of health services between 2004 and 2006 [7].

## MCH and Programmes in Adjacent Cross-Border Areas

### Northern Pakistan.

In the Northern Areas of Pakistan, which is a disputed territory without strong local governmental institutions, currently afflicted with sectarian tensions, and only accessible by road and air when the weather is favourable, public sector services have been and remain too limited to reach the great majority of the remote and dispersed rural communities. Over the past 20 years, AKDN has developed a three-tiered health system similar to the one introduced in Afghan Badakhshan. The community-based model provides outreach through a network of private, not-for-profit health facilities, linked with male and female CHWs who carry out health promotion and disease prevention activities. Communities participate in and contribute to the establishment of the health facilities, provide land and labour, and pay (limited) user fees.

Since the 1980s, there have been important improvements in MCH indicators. The community nurse-midwife (called a Lady Health Visitor) plays a key role in the system [8]. These women are the first-level health professionals with MCH skills and provide the community health workers with continuing education and supervision, perform MCH care including deliveries both at home and in the health centres, and form the link between village-level primary health care and referral health services [9]. Considerable attention is given to continuing education and supervision, and to ensuring that there is a good enabling environment with correct coordination and organisation of services, including supplies, training, and communications. The communities, through local health committees, actively support the safety and well-being of the community midwives. To ensure that referrals are timely, the community's capacity to manage complications and to respond to emergencies has been addressed simultaneously with efforts to improve the quality of facility-based health services.

### Tajikistan.

The Soviet health system contributed successfully to achieving relatively good health status for the communities in Gorno-Badakhshan. At the same time, however, the system in Gorno-Badakhshan had an excess number of hospital beds and staff (with an estimated 9.1 hospital beds, 2.1 doctors, and 16.4 nurses per 1,000 population) (see Table 1).

Health facilities and services, once widely available and generously financed by Moscow, became almost non-functional following the breakup of the Soviet Union, the cessation of subsidies, and the outbreak of civil war (1992–1997). All public sectors, including health care, were expected to make a transition from a state-run to a more market-oriented system. Over time, the government recognised the need to make structural changes and defined a health reform strategy. To achieve its vision of a sustainable, cost-effective health system accessible to all, the government intends to promote effective public health measures: enhancing primary health care by developing a family medicine speciality; reducing duplication and increasing efficiency in the hospital system; building the capacity of the health professionals; and involving the community in developing and governing the system [10].

MCH interventions in the programme include birth preparedness through the provision of community-level training and upgrading and equipping of health facilities; newborn care and treatment through awareness raising and training on neonatal care and complications; maternal and young child nutrition through trainings on breast-feeding, child development and complementary feeding, support for routine growth monitoring and research, testing the feasibility and effectiveness of adopting a home-based food fortification approach that uses “sprinkles” (sachets containing a combination of vitamins, folic acid, iron, and zinc); and household-level water supply and sanitation through health and hygiene promotion by CHWs.

## MCH Indicators, Health Systems, and Difficult Environments

The experiences described above are not formal studies, and it is difficult to attribute impact to specific interventions. Moreover, it would be incorrect to attribute the reduction in maternal or infant mortality in the Northern Areas of Pakistan solely to health system interventions. Factors such as a better-educated population, empowerment of women, and improved transportation infrastructure are undoubtedly important. One can argue that a reduction of maternal and infant mortality reflects the combined and complex interplay of factors within health systems, but also in the political, economic, social, and cultural spheres. Women's work load, nutritional status, education, access to productive resources, and social standing in society are influenced by context-specific gender relations as well as levels and distribution of economic resources. These factors interact with access to care of different quality in the three areas discussed in this paper and produce very different maternal and infant/child health outcomes.

Reducing maternal and child mortality is an immense challenge. This is especially so in difficult environments, defined as those where the state is unable to harness domestic and international resources effectively to reduce poverty [11]. Projections indicate that the targets of the global Millennium Development Goals, including the target to reduce the maternal mortality ratio by three quarters and the under-five mortality rate by two thirds, cannot be met, regardless of how much progress is made in other poor, but well-governed environments, unless substantial improvement takes place in the many areas of the world that are regarded as “difficult environments” [12].

Developing human resources, introducing quality-improvement interventions, and implementing strategies for scaling-up and sustaining interventions have been identified as the top priorities for strengthening health systems in the Northern Areas of Pakistan over the last 20 years, and more recently in Gorno-Badakhshan and Afghan Badakhshan. Going to scale and sustaining MCH programmes in difficult environments is rarely achieved. For example, after 20 years of implementation, the cost-recovery of the Pakistan Northern Areas health programme, with its primary objectives of reducing maternal and child mortality, stands at approximately 50%.

The programme in Afghanistan is implemented within the framework of performance-based partnership agreements, a “contracting-out model” [13,14]. International donors provide funding on a standard per capita basis. In addition to donor funding, modest user fees for services were charged until recently, albeit with many exemptions to protect the poor. Fees and exemption rules were set by the village health committee that also decided on the use of the income. These fees recovered an estimated 4% of total operational costs.

In the mid-term, even with an improving local economy, going to scale will depend on securing external funding. At present, Afghanistan does not have enough resources, partly because international donor agencies are not committing financial support beyond the short-term. A stronger economic base might be attained by the compound/multiplier effect of the integrated programming and area development approach that is being put in place; still, this will take time.

In Tajikistan and Gorno-Badakhshan, the transition from a state-run to a more market-oriented system called for a shift from centralised public financing, with health care free at the point of delivery, to a more cost-effective system based on a mixed financing model underpinned by social health insurance. In practice, however, the transition has not happened as predicted; the shift was imposed from outside and took place suddenly, leading to a breakdown in the health infrastructure. Financial shortages have triggered a huge increase in out-of-pocket payments, including informal payments to health professionals, who saw their salaries drop to levels below US$1 per day, and a concurrent decrease in coverage for the population [15]. A carefully planned health insurance schedule that drives the health reform, has systems for monitoring quality of care, has greater involvement of community and civil society organisations in decision making, and is designed to protect and promote the health status of the most vulnerable in Tajik society, i.e., women of reproductive age and children under five years of age, might be a way forward [16]. However, this will take time and will require all stakeholders to agree on: (a) how contracting relationships will work; (b) how to develop regulations that govern these relationships; and (c) how to strengthen capacity to implement rules designed to encourage innovation and reduce risk of failure.

**Figure 1:**
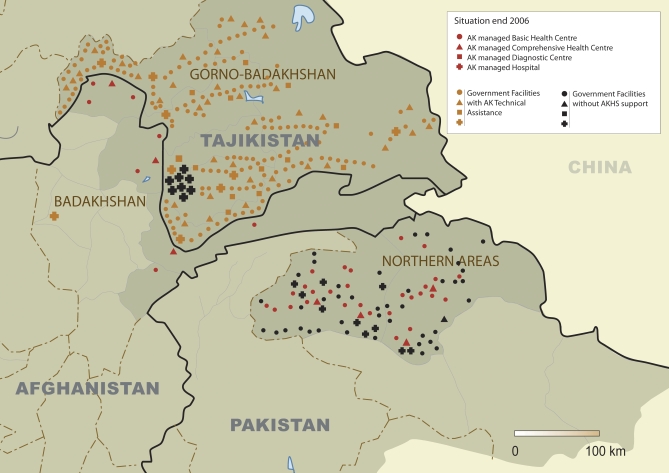
Health Facilities in the Border Districts of Afghan Badakhshan, the Northern Areas of Pakistan, and Gorno-Badakhshan in Tajikistan

One additional strategy that deserves more attention is an approach based on “cross-border health care”. Traditionally the borders were porous and people crossed easily and often to visit markets and trade; marriages across the border were common. This changed from the 1930s onwards, when the Tajik border became tightly controlled by Russian border guards and the borders were virtually closed. Following the collapse of the Soviet Union, and with the opening of three bridges over the Panji river since 2003 at the Afghan and the Tajik Gorno-Badakhshan border, a start has been made to restore some of the former practices that served local community needs. However, to date, MCH programmes in each location have been addressed within the capacities of the states and the civil society institutions. These efforts have produced very different results because the capacities of the key actors vary considerably. Enhancing capacities is a long-term effort but one that must be started immediately, and efforts to promote cross-border collaboration have the potential to lead to quick but sustainable improvements. Our experience shows that recruiting and posting health professionals (mainly MCH specialists) from Gorno-Badakhshan and the Northern Areas of Pakistan, who speak the same language and share a common cultural background, to Afghan Badakhshan is possible and acceptable to both health professionals and the local communities. It has also been possible to get some critically ill patients from Afghan Badakhshan treated in Tajik hospitals (Figure 1 presents the positioning of health facilities at different levels in the three geographical areas), and for policy makers from Gorno-Badakhshan and Afghan Badakhshan to make study tours to the Northern Areas of Pakistan in order to observe its primary health care system. As confirmed by focus group discussions and in-depth interviews held with health professionals, patients, border authorities, and policy makers, cross-border health care is achievable, but only if it is well-regulated and the regulations are enforced.

A main challenge is to overcome the lack of trust at the level of the central governments, mainly related to cross-border security and narcotics smuggling issues. The recent increase in insecurity in Afghanistan has resulted in an increase in the number of days of complete closure of the borders, and we are aware of maternal deaths in the Afghanistan border districts of patients who would have had an excellent chance of survival and full recovery if they had been able to cross the border. However, it could be argued that defining an effective cross-border health policy could be a first important step towards building trust. Measures that haven't been addressed sufficiently but could greatly increase the chances for success, and were recommended by participants in our qualitative research work include simplification of the border crossing procedures, setting up transparent costing and pricing at full cost-recovery level of all interventions with agreed financing mechanisms, and adequate communication of the potential benefits for all stakeholders in the different countries.

A cross-border health policy offers the potential to release human and financial resources in all three states, contributing to more rapid progress than can be imagined by the “conventional routes” because the approach maximises the use of existing resources. Although not easy to implement, experiences elsewhere have shown that “regulated” movement of health professionals and patients is possible; moreover, it can improve health care by making use of different capabilities of health care services in different countries [17].

## Abbreviations

AKDN: Aga Khan Development Network
CHW: community health worker
MCH: maternal and child health
